# Language Matters: Exploring Preferred Terms for Diverse Populations

**DOI:** 10.1177/23333936241275266

**Published:** 2024-09-03

**Authors:** Higinio Fernandez-Sanchez, Emmanuel Akwasi Marfo, Diane Santa Maria, Mercy Mumba

**Affiliations:** 1 The University of Texas Health Science Center at Houston, USA; 2 University of Alberta, Edmonton, Canada; 3 The University of Alabama, Tuscaloosa, USA

**Keywords:** diversity, equity, inclusion, language, social justice, social marginalization

## Abstract

This article explores the significance of employing preferred terms and inclusive language in research practices concerning diverse populations. It highlights how inappropriate terminology can lead to labeling, stereotyping, and stigma, particularly for equity-denied groups. The study aimed to identify and analyze terminology preferences for diverse communities by major international organizations. Through a systematic environmental scan methodology, data were collected from 12 prominent organizations. The results indicate a concerted effort toward adopting inclusive language, with organizations favoring respectful and accurate terminology. For instance, terms like “people made vulnerable by systemic inequities” and “migrant workers” were preferred over outdated or stigmatizing alternatives. The discussion emphasizes the importance of identifying conflicting terms and trends in terminology preferences over time. We recommend prioritizing the use of preferred terms to promote respectful and accurate discourse, with a focus on person-centered language. Ultimately, the findings underscore the critical role of language in shaping perceptions and attitudes toward diverse communities, and advocate for continued efforts to promote inclusivity and equity in research, policy, and practice.

## Introduction

Language and terms used for describing some populations can contribute to problematic outcomes from research practices and knowledge dissemination and negatively impact equity-denied groups, which include populations who face barriers to accessing resources and opportunities that are available to other members of the society because of systemic discrimination and disadvantage (Government of Canada, 2024). Past research involving various populations, particularly equity-denied members of society from different contexts, showed how inappropriate use of terms to describe research participants contributes to labeling, ordering, stereotyping, and stigma (Di Pietro & Illes, 2016; Grover et al., 2020; Linda et al., 2009; Yang et al., 2015). There is much awareness of smothered constructs for diverse social identities and locations and the surrounding sensitivities and controversies about the use of those terms in the scientific literature, education, and practices Flanagin, Frey, and Christiansen (2021), given that most social identities, including race and ethnicity are socially-constructed and may be context-specific rooted in the socio-historical and political environments (Crenshaw, 2018). For instance, Almaguer (1998) work highlighted how the sexual behavior and sexual identity of Chicano gay men are shaped by distinct American-European and Latin American sexual systems and how one embraces being “a gay.” Thus, researchers tend to inaccurately describe participants or fail to categorize some populations who may not fit into groups assigned by researchers based on dominant-Eurocentric Anglo-Saxon definitions and labels. This problem is more profound in ethnocultural contexts like Australia, Brazil, Canada, France, New Zealand, the United Kingdom, and the United States, where discussions about using preferred and inclusive language to describe populations are ongoing (Flanagin, Frey, & Christiansen, 2021; *The New York Times*, 2021) and recommendations to support equity and inclusion have been developed by many organizations (Canadian Institute for Health Information, 2022).

The use of preferred terms and inclusive language for describing populations in research demonstrates attentiveness to the potential positive and negative impact of research on a particular population or group (CDC, 2022a). Those preferred terms display respect for communities involved in the research (Flanagin, Frey, & Christiansen, 2021), eliminate biases and stigma, and can easily reach and be used by intended audiences (CDC, 2022a). Owing to this essence of preferred languages, many journals and organizations Flanagin, Frey, and Christiansen (2021), including American Psychological Association (APA, 2023a), American Cancer Society (2023), American Medical Association (AMA, 2021a), and the Canadian Institute for Health Information (2022) have progressively developed policy statements, frameworks, and guidelines for reporting identities (e.g., gender, race, ethnicity, disease conditions, disabilities, incarceration, and sexual orientation) among diverse communities in research planning and dissemination that is continuously updated. Likewise, many publishers, including SAGE (2024), Elsevier (2024), Taylor & Francis (2024), and Wiley (n.d.) support Equity, Diversity, and Inclusivity commitments by encouraging diverse and equitable language and referencing. Additionally, international organizations, including the World Health Organization (WHO) have implemented a strategic communications framework for an inclusive and respectful messaging and communication with and about diverse populations that is periodically updated (WHO, 2017). Yet, there remain knowledge gaps in how major international organizations use or apply preferred terminologies for diverse populations and communities in their activities, including research, policy, and knowledge dissemination, justifying a need for more research on this topic.

Obtaining a global picture of the preferred terminologies used by major international organizations will generate insights into gaps and inconsistencies in terminology use and offer opportunities to suggest recommendations for consistent, respectful, and culturally appropriate terminologies for these organizations. Therefore, the purpose of this study was to identify and analyze the terminology preferences for diverse communities and populations applied by major international organizations.

## Methods

### Design

We employed a systematic environmental scan (e-scan) methodology to identify non-harming, preferred terms for population groups and communities (Shahid & Turin, 2018). Our approach followed these six steps:

#### Step I: Research Question

We formulated our research question using the Population, Concept, and Context framework (Parker et al., 2021): “What are the preferred terms (Concept) for population groups and communities (Population) as identified by international organizations (Context)?”

#### Step II: Selection Criteria

We included all available English-language information on preferred terms for population groups and communities from the official web pages of international organizations.

#### Step III: Search Strategy and Data Sources

A trained research assistant conducted a systematic search of the webpages of 12 prominent international organizations Such as the United Nations (UN, 2023), International Labor Organization (ILO, 2020), UN Women (n.d.), International Organization for Migration (IOM, 2024), United Nations Development Program (UNDP, 2014), Centers for Disease Control and Prevention (CDC, 2022a), APA (2023b), Joint United Nations Program on HIV/AIDS (UNAIDS, 2015), AMA (2021b), United Nations High Commissioner for Refugees (UNHCR, 2021), United Nations Educational, Scientific and Cultural Organization (UNESCO, 1999), and the WHO (2023). The 12 international organizations selected for review were chosen based on their influential roles in shaping global standards and policies related to our research focus on preferred terms for population groups and communities. Each organization contributes expertise in areas crucial to defining terminology, including migration, health, gender equality, and labor standards. Their inclusion ensured a comprehensive examination of preferred terms across diverse sectors, enriching the breadth, and relevance of our study findings.

#### Step IV: Screen and Selection

Our search strategy used terms from the National Library of Medicine’s Medical Subject Headings (MeSH) database, such as “vulnerable populations” and “minority groups.” Additional search terms included “population demographics,” “ethnic groups,” “gender identity,” “sexual orientation,” “socioeconomic status,” “cultural communities,” “LGBTQ+ communities,” “immigrant populations,” “indigenous communities,” “disabled or differently-abled individuals,” “religious groups,” “urban communities,” “rural populations,” and “refugee communities.” We also included terms related to preferred language and terminology, such as “inclusive language” and “culturally sensitive terminology.”

#### Step V: Data Extraction

All relevant information was transcribed into a structured Excel spreadsheet by a trained research assistant. Data captured included: (a) terminology variations for different population groups, (b) contextual information accompanying preferred terms, (c) citations and source details, and (d) any nuances in the usage of specific terms.

#### Step VI: Analysis and Synthesis

It involved conducting content analysis (Krippendorff, 2019) on the collected data to discern recurring themes and patterns in the preferred terms adopted by the selected international organizations. The research team meticulously grouped and categorized the identified terms into clusters representing diverse population groups and communities. To ensure comprehensive evaluation, an expert panel comprising specialists in HIV, migration, homelessness, substance use, LGBTQ+ issues, and older adult populations was convened. Each panelist brought unique expertise to review the findings critically. Their invaluable feedback was thoughtfully integrated to enrich the depth and relevance of this project.

## Results

Our study involved examining the web pages of 12 international organizations. We could not locate the preferred terms page for two organizations; however, they did have a Glossary of Terms (UNESCO, 2023; WHO, 2021). Among the population groups identified, we found terminology guidance for 10 groups: migrants, persons with disabilities, persons living with HIV, people experiencing homelessness, persons in custody, persons who use drugs, people with mental health disorders, older adults, persons of various racial identities, and persons with an LGBTQ+ identity. Additionally, one organization provided guidance for people made vulnerable in general, and another organization that focused on gender-inclusive language (see Table 1).

**Table 1.** Organizations and Populations Addressed.

Organizations	Populations addressed
UN	People with disability
ILO	Migrant populations
UN Women	Gender-inclusive language
IOM	Migrant populations
UNDP	Multiple populations
CDC	Multiple populations
APA	Multiple populations
UNAIDS	Multiple populations, primarily people with HIV
AMA	Multiple populations
UNHCR	People with an LGBTQ+ identity

Inclusive language guides used by organizations typically feature a structured format designed to promote respectful and accurate communication across diverse contexts. They begin with an introduction emphasizing the importance of inclusivity in language use, followed by definitions of key terms such as “inclusive language” and “preferred terms.” These guides provide specific guidelines for language use, advocating for person-centered and non-stigmatizing language choices. Practical examples and scenarios illustrate these principles, helping users apply them effectively. For instance, the CDC (2022b) uses “Instead of this (lists the terms we should avoid) and Try this (lists the terms we should use).” Additional resources and acknowledgments of contributors may be included to support ongoing learning and guide updates. Overall, these guides serve as comprehensive tools to foster environments that respect and reflect the diversity of individuals and communities.

The preferred terms for population groups, as outlined in Table 2, reveal nuanced approaches aimed at promoting dignity, respect, and inclusivity while addressing the diverse needs and identities within various communities. For instance, when referring to migrants, preferred terms such as “Migrant worker” and “Undocumented migrant” highlight the importance of recognizing the different circumstances and statuses among migrant populations (AMA, n.d.; APA, 2023b; ILO, 2020; IOM, 2019). Similarly, descriptors for persons with disabilities emphasize person-first language and specific descriptors based on the type of disability or impairment, reflecting a respectful and inclusive approach (AMA Manual of Style Committee, 2020; APA, 2023b; CDC, 2022b; UN, 2023; UNAIDS, 2015). In the context of persons living with HIV, preferred terms focus on person-first language while also addressing treatment and advocacy aspects, showcasing a supportive and empowering stance (APA, 2023b; UNAIDS, 2015; UNDP, 2014).

**Table 2.** Preferred Terms for Population Groups.

Population groups	Organizations	Preferred terms
Migrants	AMA (2021b); APA (2023b); ILO (2020); ILO (2022); ILO (n.d.); IOM, (2019)	Migrant worker Undocumented migrant Migrant with irregular status Labor migration Mobility Movement Domestic worker Sex work/sex worker Forced labor Labor exploitation Human trafficking Forced marriage People with undocumented status Mixed-status households Immigrant Migrant People who are seeking asylum Refugee or refugee populations Non-U.S.-born persons/foreign-born persons Economic migrant (only in specific contexts) Temporary Foreign worker (if legally employed) International migration Mobile populations Survivors of human trafficking Victims of forced marriage People seeking refuge Displaced persons
Persons with disability	AMA (2021b); APA (2023b); CDC (2022b); UN (2023); UNAIDS (2015)	Person with disability Person with [type of impairment] Persons with disabilities People with disabilities (only in Easy Read documents, informal text, and oral speech) Person without disability Have [disability/impairment/condition] Person with an intellectual disability Person with a psychosocial disability Deaf person Person with hearing loss Blind person Person with a vision/visual disability Person with low vision Person with a physical disability Person who uses a wheelchair Person with a mobility disability Person using a mobility device Person with achondroplasia (only if the person has this condition) Person with Down syndrome Person with albinism Person affected by leprosy Person who uses a communication device Person who uses an alternative method of communication Parking reserved for persons with disabilities Accessible bathroom Child with a congenital disability Child with a birth impairment
Persons living with HIV	APA (2023b); UNAIDS (2015); UNDP (2014)	Individuals affected by HIV People affected by AIDS Person with HIV HIV-positive individual HIV and AIDS survivor Person on HIV treatment Person living with HIV-related conditions Person living with HIV who is undetectable Person living with HIV who is virally suppressed HIV advocate Person with an HIV diagnosis HIV community member People with HIV and their families Person managing HIV/AIDS Safer sex Intimate partner transmission
People experiencing homelessness	AMA (n.d.); CDC (2022b); UNDP (2014)	People experiencing homelessness Persons experiencing unstable housing/housing insecurity/persons who are not securely housed People experiencing unsheltered homelessness Clients/guests who are accessing homeless services People without housing People experiencing unsheltered People experiencing homelessness People experiencing housing or food insecurity People experiencing housing insecurity or food insecurity Individuals experiencing homelessness People without stable housing Individuals facing housing insecurity People living on the streets Those experiencing housing instability People in transitional housing Individuals experiencing housing crisis
Persons in custody	CDC (2022b)	People/persons who are incarcerated or detained (often used for shorter jail stays, for youth in detention facilities, or for other persons awaiting immigration proceedings in detention facilities) Partner/child of an incarcerated person Persons in pre-trial or with charge People who were formerly incarcerated Persons on parole or probation Persons detained by or under the custody of (specify agency) (e.g., U.S. Immigration and Customs Enforcement [ICE] or other agencies) Individuals who are incarcerated People in custody Those in detention facilities Persons under correctional supervision Individuals in the justice system People in confinement Individuals in the prison system
Persons who use drugs	APA (2023b); CDC (2022b); UNAIDS (2015)	Persons who use drugs/people who inject drugs Persons with alcohol use disorder Persons in recovery from substance use/alcohol disorder Persons taking/prescribed medications for opioid use disorder (MOUD) Persons who returned to use Person who uses drugs Person who injects drugs Person with substance use disorder Person in recovery from substance use/alcohol disorder Person taking/prescribed medications for opioid use disorder (MOUD) Person who returned to use Person who smokes Individuals who use substances People with substance use disorder Persons with substance dependence Those struggling with substance abuse Individuals with a history of substance use People with substance misuse issues Persons with substance dependency Individuals with a history of drug use Those experiencing drug addiction People managing substance use challenges
Populations made vulnerable	AMA (n.d.); APA (2023b); CDC (2022b); UNAIDS (2015); UNDP (2014)	People who are underserved by. . . People who are medically underserved People who are uninsured/people who are underinsured/people who do not have health insurance People with lower incomes People/households with incomes below the federal poverty level People with self-reported income in the lowest income bracket (if income brackets are defined) People experiencing poverty (do not use “underserved” when meaning low SES) People who live/work in settings that put them at increased/higher susceptibility of becoming exposed to hazards Communities of color Populations that are minoritized Individuals from underrepresented groups Communities that have been historically marginalized Individuals with marginalized identities Developing countries People who live in rural/sparsely populated areas Residents/populations of rural areas Residents of communities made vulnerable Groups that have been economically/ socially marginalized Groups that have been historically marginalized or made vulnerable Groups that are struggling against economic marginalization Communities that are underserved by/with limited access to (specific service/resource) Groups experiencing disadvantage because of (reason) Groups experiencing disproportionate prevalence/rates of (condition) (People from) racial and ethnic groups (People from) racial and ethnic minority groups (People from) sexual/gender/linguistic/religious groups (People with/living with) mobility/cognitive/vision/hearing/independent living/self-care disabilities
		Engage/prioritize/collaborate with/serve (population of focus) Consider the needs of/Tailor to the needs of (population of focus) Communities/populations of focus Intended audience Eliminate (issue/disease) Key populations Priority populations Engage, involve, focus, designed for and by
People with mental health disorders	APA (2023b); CDC (2022b)	People with a pre-existing mental health disorder People with a pre-existing behavioral health disorder People with a diagnosis of a mental illness/mental health disorder/behavioral health disorder Psychiatric hospital/facility Person living with a mental illness Person with a preexisting mental health disorder Person with a preexisting behavioral health disorder Person with a diagnosis of a mental illness/mental health disorder/behavioral health disorder Individuals living with mental illness Those experiencing mental health challenges Individuals managing mental health disorders People facing mental health issues Persons with psychiatric conditions Those living with emotional difficulties Individuals with psychological conditions People coping with mental health concerns Persons navigating mental health disorders
Older adults	APA (2022b); CDC (2023b)	Persons aged [numeric age group] (e.g., persons aged 55–64 years) Elders when referring to older adults in a cultural context Elderly or frail elderly when referring to older adults in a specific clinical context Elderly individuals Older persons Aging adults Retirees Older population Elderly community members Aging population Elderly residents
Persons of different racial identities	APA (2022); CDC (2023)	American Indian or Alaska Native persons/communities/populations Asian persons Black or African American persons; Black persons Native Hawaiian persons Pacific Islander persons White persons People who identify with more than one race; people of more than one race; persons of multiple races Hispanic or Latino persons African American/Black Nigerian, Kenyan, Jamaican, Bahamian, Puerto Rican, or Panamanian; in these cases, use “Black.” Asian/Asian American Refer to the specific nation or region of origin, when possible, for example, “Asian origin” may be divided regionally into South Asian, East Asian, and Southeast Asian BIPOC (Black, Indigenous, and people of color) use specific terms when referring to different racial and ethnic groups. When such a level of specificity is not possible, instead of “BIPOC” use alternative terms, such as “people/ persons of color” and “communities of color.” People from various ethnic groups
		Persons of different racial identities Individuals from diverse racial and ethnic backgrounds People of color Racially diverse individuals Ethnic community members Multicultural populations Racially and ethnically diverse communities
Persons with an LGBTQ+ identity	APA (2023b); CDC (2022b); UNHCR (2021)	- LGBTQ (or LGBTQIA or LGBTQ+ or LGBTQIA2) Lesbian, gay, or bisexual (when referring to self-identified sexual orientation) MSM (men who have sex with men) Queer Pansexual Asexual Transgender Assigned male/female at birth Designated male/female at birth Gender non-conforming Two-spirit Non-binary Genderqueer Gender diverse People/person with intersex traits Singular they or their He/she/they Transgender person Transgender people Trans and gender nonbinary folks or folx Queer LGBTQ+ LGBTQ+, LGBTQIIA+, etc. Queer community members Individuals with diverse sexual orientations and gender identities LGBTQ+ people Members of the LGBTQ+ community Gender and sexual minority individuals Queer and transgender individuals LGBTQ+ community members People with diverse gender expressions and sexual orientations Individuals with non-binary or fluid identities
Gender-inclusive	UN Women (n.d.)	People, humanity, human beings, humankind, women and men Representatives, business community, business people Chair, chairperson, head Legislator, congressional representative, parliamentarian All Human induced disaster Police officer Flight attendant First-year student Owner Layperson, average person Partners, spouses Cleaner Nurse Doctor Politician

The preferred terms for people experiencing homelessness encompass a range of descriptors that highlight housing insecurity and the diverse situations faced by individuals without stable housing (AMA, n.d.; CDC, 2022b; UNDP, 2014). Similarly, descriptors for persons in custody emphasize respectful language and avoidance of stigmatizing terminology, recognizing the dignity of individuals within the criminal justice system(CDC, 2022b). The preferred terms for persons who use drugs prioritize person-first language and emphasize recovery and support, steering away from stigmatizing language associated with substance use disorder (APA, 2023b; CDC, 2022b; UNAIDS, 2015).

Our findings highlight preferred terms for populations made vulnerable, underscoring the importance of addressing disparities in access to resources and services based on socioeconomic status, race, ethnicity, and other factors (AMA, n.d.; APA, 2023b; CDC, 2022b; UNAIDS, 2015; UNDP, 2014). Furthermore, preferred terms for persons of different racial identities and persons with an LGBTQ+ identity prioritize inclusivity, specificity, and respect for diverse identities, fostering an environment of acceptance and understanding (APA, 2023b; CDC, 2022a; UNHCR, 2021).

## Discussion

The findings from the environmental scan provide valuable insights into the evolving landscape of terminology preferences for diverse communities and populations as identified by major international organizations. The discussion of these findings highlights several important considerations regarding terminology usage, including the significance of adopting preferred terms for inclusive and respectful discourse.

Our findings highlight conflicting or controversial terms in understanding the potential impact of language on diverse population groups. For instance, the use of terms like “minority groups” can perpetuate a sense of otherness or marginalization, reinforcing stereotypes and inequalities (APA, 2023a). Additionally, terms defined solely based on numerical size, such as “minority,” may fail to capture the full extent of power dynamics and systemic discrimination experienced by these groups (Flanagin, Frey, & Christiansen, 2021). Instead, we suggest that adopting terms like “communities made vulnerable” or “populations marginalized by . . .” centers the experiences and identities of the people involved, promoting a more respectful and accurate portrayal. However, Andoh (2022) and Santiago et al. (2021) emphasized that inclusive language entails recognizing power dynamics, embracing diversity, and creating environments conducive to genuine self-expression. This process involves listening to various groups’ self-identification, respecting their language preferences (e.g., person-first, identity-first), confronting biases, and remaining open to ongoing growth and comprehension.

The consideration of sex and gender is paramount when discussing inclusive language and preferred terms. Sex and gender identity influence how individuals experience and express themselves, and the terminology used can significantly impact their well-being and recognition in society (Borger, 2023). In our scan, we observed that terms such as “gender diverse individuals” and “people of all genders” are increasingly preferred over binary and potentially exclusionary terms. These preferred terms acknowledge the spectrum of gender identities beyond the binary categories of women and men, and girls and boys, fostering inclusivity and respect (Springer, 2022). It is crucial for researchers, policymakers, and practitioners to employ gender-sensitive language that respects individuals’ self-identification and promotes equity (Rioux et al., 2022). Using inclusive language in relation to sex and gender not only validates the identities of those often marginalized but also challenges societal norms that perpetuate gender biases and discrimination.

The scan also revealed notable trends or shifts in terminology preferences over time, reflecting a growing recognition of the importance of inclusive language. Major international organizations included in this work have demonstrated a commitment to adopting respectful and accurate terminology when referring to diverse populations. This shift toward inclusive language aligns with broader efforts to promote diversity, equity, and inclusion in research, policy, and practice. For instance, Flanagin, Frey, Christiansen, and Bauchner (2021) emphasized the ongoing revisions to the *AMA Manual of Style*’s section on inclusive language, specifically focusing on terms related to race and ethnicity. We recommend that experts across various fields, including HIV, migration, and LGBTQ+ issues, not only employ preferred terms and phrases in their work but also promote efforts to advocate for inclusive language that is less harmful to the population groups they serve.

While our e-scan reveals that many guidelines have been developed to promote the use of inclusive language, there is a significant gap between these guidelines and their practical application. For instance, the National Institutes of Health (NIH, 2023a) advocates for inclusive terminology yet frequently uses terms such as “vulnerable populations” and “underserved populations” in their funding opportunity announcements. We believe that this inconsistency often arises from unconscious biases rather than intentional disregard for inclusivity. It highlights a crucial need for organizations to not only establish but rigorously implement inclusive language policies. We advocate for the consistent use of terms like “populations made vulnerable” to accurately reflect systemic inequities and avoid perpetuating stigma. Adhering to inclusive language is essential in promoting respect, equity, and a more accurate representation of diverse communities. As authors, we are also constantly vigilant in checking our own language use to ensure it aligns with these principles. It is imperative that organizations bridge the gap between policy and practice to foster truly inclusive and respectful discourse.

Based on these findings, international organizations, researchers, policymakers, community leaders, and the general population should prioritize the use of preferred terms for selecting population groups and communities in their discourse. We recommend a change in the way we employ the terms “vulnerable,” “marginalized,” “oppressed,” “hard to reach,” “underprivileged,” and “underserved.” Instead, we advocate for being as specific as possible. For example, using phrases like “communities marginalized by systemic inequalities” or “populations made vulnerable by historic discrimination and colonization” offers clarity and acknowledges the underlying factors contributing to their circumstances (Garrett & Altman, 2024). We encourage individuals to regularly check the websites of major organizations that focus on specific areas to stay updated on preferred terminology. Additionally, we recommend that whenever in doubt, starting with the word “person” can serve as a guiding principle, emphasizing the importance of person-centered language and respectful communication. For instance, phrases like “people with disabilities” or “people with HIV” acknowledge the individuality and dignity of each person while avoiding stigmatizing or dehumanizing language (NIH, 2023b).

In discussing the importance of inclusive language, we draw from Likis (2021) and Bares et al. (2023), who emphasized the significance of language choice in conveying respect and promoting equity. Likis (2021) provided recommendations for inclusive language alternatives in various domains, such as race and ethnicity, socioeconomic status, sex and gender, sexual orientation, age, and ability and disability. Bares et al. (2023) advocated for the use of person-first language in scientific discourse as a means of respecting individuals and avoiding stigmatization based on medical conditions. We recommend that organizations, journals, policymakers, researchers, and the broader community refrain from listing harmful and offensive terms. Doing so is unnecessary, potentially triggering, and could inadvertently reinforce their usage. Instead, we strongly advise directing attention towards the appropriate terms that should be used. Additionally, we suggest that when listing the race or the ethnicity of individuals, this should be done in alphabetical order. These recommendations align with broader efforts to prioritize the use of preferred terms for diverse population groups and communities in discourse, as advocated by international organizations and scholars (Ashwell et al., 2023; Garrett & Altman, 2024). See Appendix A for more recommendations.

Future research should consider several avenues to advance our understanding and application of preferred terms for population groups and communities. Firstly, there is a need for longitudinal studies that track changes in terminology preferences over time, reflecting evolving societal norms, and advancements in inclusivity. Secondly, comparative analyses across different regions and cultural contexts could provide valuable insights into the universality versus context-specific nature of preferred terms. Additionally, qualitative studies exploring community perspectives on language use and its impact on identity and well-being would deepen our understanding of the practical implications of terminology choices. Lastly, collaborative efforts involving academia, policymaking, and affected communities themselves could facilitate the development of comprehensive guidelines for inclusive language that resonate across diverse settings and disciplines.

## Conclusion

This research contributes to a more profound understanding of the complexities and considerations associated with the use of preferred terms for population groups and communities, with implications for policy development, communication, and the fostering of inclusive environments. This analysis sheds light on the evolving landscape of inclusive language and terminology within international contexts. The findings of this environmental scan underscore the critical role of language in shaping perceptions and attitudes toward diverse communities. By adopting inclusive terminology, international organizations can contribute to creating more equitable and inclusive societies, where all individuals are respected and valued. Moving forward, continued efforts to promote respectful and accurate language are essential for fostering understanding, empathy, and solidarity across diverse populations worldwide. Finally, guidelines and policy statements on appropriate and respectful use of inclusive language and terminologies need to be continually updated.

## Appendix A

We encourage organizations, journals, policymakers, researchers, and the broader community to:

- Advocate for the use of inclusive language in scientific discourse, promoting equity and respect across various domains such as race, ethnicity, socioeconomic status, sex, gender, sexual orientation, age, and ability.
- Actively promote efforts to advocate for inclusive language and discourage the use of harmful or offensive terms.
- Provide education and training on inclusive language usage to ensure that individuals are equipped with the knowledge and skills to communicate respectfully and effectively with diverse populations.
- Foster an environment of open dialogue and continuous learning, where individuals are encouraged to listen to various groups’ self-identification, confront biases, and remain open to ongoing growth and comprehension.
- Collaborate with communities to co-create language guidelines that reflect the preferences and needs of the populations being addressed.
- Conduct regular reviews and updates of terminology guidelines to reflect evolving language preferences and best practices in promoting inclusivity and respect.
- Encourage the adoption of preferred terms not only in written communication but also in verbal and oral discourse to ensure consistency and alignment with inclusive language principles.
- Prioritize the use of preferred terms for selecting population groups and communities in discourse, avoiding terms that can be perceived as harmful.
- Whenever in doubt, start with the word “person” as a guiding principle, emphasizing the importance of person-centered language and respectful communication.
- Regularly check the websites of major organizations that focus on specific areas to stay updated on preferred terminology.
- When listing the race or ethnicity of individuals, do so in alphabetical order to promote fairness and consistency.
- Engage in ongoing advocacy for inclusive language practices, recognizing the significance of language choice in conveying respect and promoting equity.
